# Clinical outcomes of fibular and scapular free osseous flaps in head and neck reconstruction: a retrospective comparative study

**DOI:** 10.1007/s00405-026-10299-5

**Published:** 2026-05-11

**Authors:** Vittorio Favero, Gabriele Sala, Francesca Mazzarella, Guido Bissolotti, Alessandra Ruaro, Stefano Taboni, Piergiorgio Gaudioso, Piero Nicolai, Marco Ferrari, Cesare Tiengo

**Affiliations:** 1 Department of Neuroscience, Section of Maxillofacial Surgery, University of Padova, Via Giustiniani 2, Padova, 35128 Italy; 2 Department of Neuroscience, Clinic of Plastic Surgery, University of Padova, Via Giustiniani 2, Padova, 35128 Italy; 3 Department of Neuroscience, Section of Otorhinolaryngology – Head and Neck Surgery, University of Padova, Via Giustiniani 2, Padova, 35128 Italy; 4 Unit of of Otorhinolaryngology – Head and Neck Surgery, Azienda Ospedale-Università Padova, Via Giustiniani 2, Padova, 35128 Italy

**Keywords:** Fibular flap, Scapular flap, Microvascular reconstruction, Patient-reported outcomes, Head and neck oncologic surgery

## Abstract

**Purpose:**

Osseous microvascular reconstruction of the head and neck following oncologic or osteoradionecrotic resection remains surgically challenging. The fibular free flap is the gold standard for mandibular reconstruction, whereas the scapular free flap has emerged as a viable alternative in selected scenarios.

**Methods:**

A retrospective comparative analysis was performed on 69 adults undergoing cervicofacial reconstruction with fibular (*n* = 37) or scapular (*n* = 32) osseous free flaps (2015–2025). Surgical, functional, and patient-reported outcomes (DASH, EFAS, SHI, MDADI, EORTC QLQ-H&N35, FACE-Q) were assessed.

**Results:**

Demographics and clinical profiles were similar, though scapular recipients were older. Fibular flaps predominate in mandibular, scapular in maxillary reconstructions. Fibular reconstructions used more custom plates and had longer follow-up but exhibited higher donor-site complication (24.3%) and elective revision (62.2%) rates. PROMs were comparable; extensive scapular flaps caused mild upper limb function decline.

**Conclusion:**

Scapular flaps demonstrated lower donor-site complication rates and comparable functional and patient-reported outcomes in this cohort. These findings suggest that the scapular flap represents a valuable reconstructive alternative, particularly in patients in whom fibular harvest may be less suitable.

## Introduction

Reconstruction of complex cervicofacial defects remains a cornerstone in head and neck oncologic surgery. Ablative procedures involving the mandible or maxilla, whether for malignancy, osteoradionecrosis, or aggressive benign pathology, result in significant functional and aesthetic impairments, necessitating precise reconstruction to restore speech, swallowing, mastication, and facial contour.

Preoperative planning is essential, especially when complex skeletal structures are involved. Its purpose is to precisely define the lesion extent, anatomical relationships and surgical strategy. Digital tools, especially Virtual Surgical Planning (VSP), have become standard use.

VSP overcomes limits of freehand surgery through virtual simulation, 3D modeling, and patient-specific guides and plates, improving resection accuracy, flap shaping and fixation, reducing operative time, and supporting implant rehabilitation [1, 2]. Segmental mandibular or maxillary loss severely affects function and appearance. Loss of bone support impairs mastication, swallowing, phonation, and respiration, and alters symmetry and expression [3].

Lateral mandibular defects mainly cause occlusal alteration and deviation; posterior defects may be managed with soft tissue but still impair mastication. Maxillary loss creates oro-nasal communication, dysphagia, speech changes, and, if the palate is involved, velopharyngeal insufficiency [4]. Anterior maxillary defects add midfacial collapse and upper-lip retraction [5].

Based on these issues, main reconstructive goals are [6–8]:

1. Immediate and stable wound closure. Single-stage reconstruction of mandibular and maxillary defects improves recovery and reduces psychological stress [9]. Delayed closure increases dehiscence, infection, and fistula risk, and may postpone radiotherapy beyond the optimal six-week window [10].
2. Restoration of oral function. After mandibulectomy, restoring bony continuity preserves occlusion, joint mobility, and mastication; soft tissue repair enables phonation, swallowing, and airway protection. After maxillectomy, priorities are oral-nasal separation, a stable base for speech and chewing, and prosthetic support. Orbital defects require restoration of volume, globe position, and eyelid symmetry [11].
3. Recovery of facial harmony. Re-establishing skeletal landmarks and soft tissue volume is crucial for social reintegration.

Vascularized osseous free flaps are the gold standard. The fibular free flap (FFF) described by Hidalgo [12], is preferred for mandibular reconstruction due to its long bicortical segment and implant potential but requires adequate limb vasculature and carries donor-site morbidity [13]. The scapular free flap (SFF), introduced by Swartz, provides reliable bone and soft tissue, minimal ambulation impact, and is well suited for maxillary defects due to its large multi-plane components. Harvest in lateral decubitus allows two-team surgery [13]. Despite advantages, SFF has been underused due to technical concerns and limited bone length. With VSP and patient-specific implants, its utility now approaches that of FFF, with studies showing similar outcomes and prompting further comparison.

This retrospective study compares clinical outcomes, donor-site morbidity, and patient-reported outcomes in fibular versus scapular osseous flaps [14]. The primary endpoint of the study was the incidence of donor-site complications, chosen as a clinically relevant measure of flap-related morbidity that may influence reconstructive decision-making. Secondary endpoints included overall postoperative complications, revision surgery rates, long-term recipient-site complications, and functional and patient-reported outcomes. The aim of the study was to support personalized surgical planning in complex maxillofacial reconstruction.

## Materials & methods

### Study design and setting

This was a longitudinal, retrospective, single-center study conducted at the University Hospital of Padua, involving the Departments of Maxillofacial Surgery, Otorhinolaryngology, and Plastic Surgery. Patients who underwent cervicofacial reconstruction between 2015 and 2025 were included.

#### Patients and data collection

Clinical, instrumental, and demographic data were collected from outpatient records, operative notes, and inpatient charts. The cohort included patients reconstructed with osseous free flaps: fibula (*n* = 37) or scapula (*n* = 32). Outcomes were assessed ≥ 6 months postoperatively through follow-up and validated questionnaires. All patients provided informed consent.

Recorded variables included demographics, defect site, type of resection (mandible/maxilla), classification (Jewer HCL, Brown), and flap type. Intraoperative data included soft tissue reconstruction, CAD-CAM plates, tracheostomy, transfusions, and operative time. Postoperative data comprised time to oral feeding, decannulation, hospital stay, and complications. Complications were labeled early (≤ 48 h) or late (> 48 h during hospitalization), and classified as medical or surgical (e.g., flap loss, donor-site issues). Need for revision surgery was noted. Long-term follow-up documented late events such as plate exposure, fistula, pseudoarthrosis, osteoradionecrosis, infection, and elective revisions.

####  Study endpoints

The primary endpoint of the study was the incidence of donor-site complications following fibular or scapular free flap harvest. Donor-site morbidity was selected as the main outcome because it represents a key determinant in reconstructive flap selection and has direct implications for postoperative functional recovery.

Secondary endpoints included early and late postoperative complications, need for urgent or elective revision surgery, long-term recipient-site complications (including plate exposure, fistula formation, pseudoarthrosis, and infection), postoperative recovery parameters (time to oral feeding, decannulation, and length of hospital stay), and patient-reported outcome measures (PROMs), including speech function (SHI), swallowing function (MDADI), limb function (DASH and EFAS), quality of life (EORTC QLQ-H&N35), and aesthetic satisfaction (FACE-Q).

#### Inclusion criteria

- Age > 18 years.
- Cervicofacial reconstruction with fibula or scapula osseous free flap.
- Complete clinical documentation (anamnesis, diagnostics, surgery, follow-up data).

#### Exclusion criteria

- Age < 18 years.
- Reconstruction not involving the cervicofacial region.
- Use of non-osseous or alternative free flaps.
- Incomplete records or insufficient follow-up.

#### Patient-reported outcome measures (PROMs) and classification tools

For donor-site morbidity, upper limb function was assessed with the Italian validated version of the *Disabilities of the Arm*,* Shoulder and Hand* (DASH) questionnaire, while for lower limb function the *European Foot and Ankle Society* (EFAS) score was used (EFAS Scientific Committee) [15]. Speech impairment was measured using the Italian validated *Speech Handicap Index* (SHI), swallowing with the *M.D. Anderson Dysphagia Inventory* (MDADI), quality of life with the *EORTC QLQ-H&N35* module, and aesthetic perception with the *FACE-Q Head and Neck Cancer Module – Appearance Scale*. All questionnaires were self-administered, validated, and scored according to standard guidelines, converted to a 0–100 scale for comparability, where higher scores indicated better function or greater satisfaction, except for the EORTC H&N35, where higher scores reflected greater symptom burden.

For defect description and surgical planning, the HCL classification of Jewer et al. [16] was used for mandibular defects, the Brown classification for maxillary defects [16]. In this study, only the simplified HCL system based on capital letters (H, C, L) was applied, without extensions for soft tissue involvement.

- DASH: 30-item scale (scores 0–100 (higher = greater disability) [7].
- EFAS: 6-item scale assessing pain and physical function of the lower limb after fibula harvest; scores converted to 0–100 (higher = better function).
- SHI: 30 items measuring perceived speech handicap; scores 0–100 (higher = worse impairment).
- MDADI: 20 items across functional, emotional, and physical domains, plus one global item; composite score 20–100 (higher = better swallowing function).
- EORTC QLQ-H&N35: 35 items across multiple head-and-neck–specific domains; selected subdomains relevant to this study (pain, taste/smell, social eating, social contact, dental issues, trismus, xerostomia, saliva consistency, nutritional support, weight changes). Scores 0–100 (higher = worse symptoms).
- FACE-Q Appearance Module: 10 items evaluating facial aesthetics, symmetry, scars, and satisfaction; scores 0–100 (higher = better satisfaction).

### Statistical analysis

Patient data were tabulated in Microsoft Excel and statistically processed using Jamovi (v2.6.26). Categorical variables were expressed as absolute and relative frequencies and analyzed using the chi-square (χ²) test. Distribution of continuous variables was assessed with the Shapiro–Wilk test. Parametric data were reported as mean ± standard deviation (SD) and compared using the independent-samples t-test, whereas non-parametric data were expressed as median (IQR) and analyzed with the Mann–Whitney U test. Statistical significance was set at *p* < 0.05.

Comparative analyses were performed between fibula and scapula flap cohorts. Operative time, length of stay, time to oral feeding, and time to decannulation were evaluated using the Mann–Whitney U test. In-hospital complications (early vs. late; medical vs. surgical), need for urgent or elective revision surgery, and late postoperative events were assessed with the χ² test. PROMs were analyzed according to instrument-specific characteristics: SHI, MDADI, and EORTC QLQ-H&N35 were compared using the Mann–Whitney U test, while FACE-Q Appearance scores were analyzed with the independent-samples t-test. DASH and EFAS scores were standardized on a 0–100 scale; to allow uniform interpretation, DASH scores (higher = greater disability) were inverted (100 – DASH), so that higher values consistently reflected superior function across all PROMs.

#### Multivariable analysis of key clinical endpoints

To account for baseline imbalances in reconstruction site, age, adjuvant radiotherapy, and follow-up duration, multivariable logistic regression analyses were performed for selected clinically relevant endpoints, including donor-site complications, plate exposure, fistula formation, and elective revision surgery. Each model included the following covariates: age, sex, smoking status, reconstruction site (mandible vs. maxilla), adjuvant radiotherapy, and follow-up duration. Multivariable analysis did not demonstrate any independent association between flap type and donor-site complications, fistula formation, or elective revision surgery. In contrast, plate exposure was independently associated with reconstruction site and follow-up duration, with a significantly higher likelihood of plate exposure observed in maxillary reconstructions and with longer follow-up. No additional variables were found to be independently associated with the analyzed outcomes.

## Results

A total of 73 patients underwent cervicofacial reconstruction at Padua University Hospital (2015–2025). After exclusion of one latissimus dorsi flap case and three patients with incomplete documentation, 69 patients were eligible for analysis.

### Demographics and surgical features

Of the 69 patients, 37 (53.6%) received a fibula free flap and 32 (46.4%) a scapular flap. Median age was 60 years (IQR 51–69; range 19–85), significantly lower in the fibula group (53.5 vs. 63.0 years; *p* = 0.018). The sample comprised 37 males (53.6%) and 32 females (46.4%). Smoking status was negative in 55.1%, active in 20.3%, and former in 24.6%; additional substance use was reported in 15.9%. Comorbidities were present in 53 patients (76.8%) (Tables 1 and 2).

**Table 1:** Clinical characteristics of the study population

	Fibula (*N* = 37)	Scapula (*N* = 32)	Total (*N* = 69)	*p* value
Age, years				0.018
Median (IQR)	53.5 (51–63)	63.0 (52.3–76)	60 (51–69)	
Range	19–76	19–85	19–85	
Gender				0.516
Male	18 (48.6%)	19 (59.4%)	37 (53.6%)	
Female	19 (51.4%)	13 (40.6%)	32 (46.4%)	
Smoking				0.817
Never	21 (56.8%)	17 (53.1%)	38 (55.1%)	
Current	8 (21.6%)	6 (18.8%)	14 (20.3%)	
Ex-smoker	8 (21.6%)	9 (28.1%)	17 (24.6%)	
Substance of abuse				0.947
No	31 (94.6%)	27 (84.4%)	58 (84.1%)	
Yes	6 (16.2%)	5 (15.6%)	11 (15.9%)	
Comorbidity				0.166
Absent	11 (29.7%)	5 (15.6%)	16 (23.2%)	
Present	26 (70.3%)	27 (84.4%)	53 (76.8%)	
ASA				
1	2 (5.4%)	0.0 (0.0%)	2 (2.9%)	
2	16 (43.2%)	17 (53.1%)	33 (47.8%)	
3	19 (51.4%)	15 (46.9%)	34 (49.3%)	
Diagnosis				0.149
SCC	19 (51.4%)	22 (68.8%)	41 (59.4%)	
Recurrent	3 (8.1%)	5 (15.6%)	8 (11.6%)	
Sarcoma	3 (8.1%)	3 (9.4%)	6 (8.7%)	
Recurrent	1 (2.7%)	1 (3.1%)	2 (2.9%)	
Ameloblastoma	2 (5.4%)	1 (3.1%)	3 (4.3%)	
Other Tumors	3 (8.1%)	5 (15.6%)	8 (11.6%)	
Recurrent	0 (0%)	1 (3.1%)	1 (1.4%)	
ORN	9 (24.3%)	1 (3.1%)	10 (14.5%)	
Post-extraction osteomyelitis	1 (2.7%)	0 (0%)	1 (1.4%)	
Previous Flap				0.662
Not performed	31 (83.8%)	28 (87.5%)	59 (85.5%)	
Performed	6 (16.2%)	4 (12.5%)	10 (14.5%)	
Neoadjuvant Therapy				0.121
No	33 (89.2%)	24 (75.0%)	57 (82.6%)	
Yes	4 (10.8%)	8 (25.0%)	12 (17.4%)	
Neoadjuvant CT	4 (10.8%)	8 (25%)	11 (15.9%)	
Neoadjuvant RT	0 (0%)	2 (6.2%)	2 (2.9%)	
Adjuvant Therapy				0.005
No	19 (51.4%)	6 (18.8%)	25 (36.2%)	
Yes	18 (48.6%)	26 (81.2%)	44 (63.8%)	
Adjuvant CT	7 (18.9%)	5 (15.6%)	12 (17.4%)	
Adjuvant RT	16 (43.2%)	25 (78.1%)	41 (59.4%)	
Follow up, months				0.004
Median IQR	14 (7–36)	7.5 (6–17.3.3)	11 (7–26)	
Range	6–99	6–30	6–99

**Table 2:** Intraoperative and surgical characteristics

	Fibula (*N* = 37)	Scapula (*N* = 32)	Total (*N* = 69)	*p* value
Defect Site				< 0.001
Maxilla	5 (13.5%)	21 (65.6%)	26 (37.7%)	
Mandible	32 (86.5%)	10 (31.2%)	42 (60.9%)	
Both	0 (0%)	1 (3.1%)	1 (1.4%)	
Custom made				< 0.001
No	8 (21.6%)	26 (81.2%)	34 (49.3%)	
Yes	29 (78.4%)	6 (18.8%)	35 (50.7%)	
Tracheostomy				0.503
Not performed	4 (10.8%)	2 (6.2%)	6 (8.7%)	
Performed	33 (89.2%)	30 (93.8%)	63 (91.3%)	
Intraoperative transfusions	7 (18.9%)	3 (9.4%)	10 (14.5%)	0.261
Not required	30 (81.1%)	29 (90.6%)	59 (85.5%)	
Required	7 (18.9%)	3 (9.4%)	10 (14.5%)	
Surgical Time, hours				0.608
Mean ± SD	11.7 ± 2.2	11.9 ± 2.1	11.8 ± 2.1	
Range	8.2–17.2	8.5–16.5	8.2–17.2	

#### Intraoperative findings

Reconstruction sites differed significantly between groups (*p* < 0.001): fibula flaps were mainly used for mandibular defects (86.5%), while scapular flaps predominated in maxillary defects (65.6%). One patient presented with combined maxillary–mandibular involvement. Use of CAD/CAM customized plates was significantly higher in the fibula group (78.4% vs. 18.8%, *p* < 0.001). Tracheostomy was performed in 91.3% of cases, with no group differences. Intraoperative transfusions were required in 14.5%, with a non-significant trend toward higher frequency in the fibula group. Mean operative time was 11.8 ± 2.1 h, with no significant difference between groups (Tables 3 and 4).

**Table 3:** Surgical revisions following free flap reconstruction

	Fibula (*N* = 37)	Scapula (*N* = 32)	Total (*N* = 69)	*p* value
Revision surgery				0.044
Not performed	13.0 (35.1%)	19.0 (59.4%)	32.0 (46.4%)	
Performed	24.0 (64.9%)	13.0 (40.6%)	37.0 (53.6%)	
Urgent	6.0 (16.2%)	8.0 (25.0%)	14.0 (20.3%)	0.366
Elective	23.0 (62.2%)	10.0 (31.2%)	33.0 (47.8%)	0.010

**Table 4:** DASH and EFAS scoring

	*N*	Mean ± SD	Median	Range	IC 95%
DASH	23	31.5 ± 23.8	31.0	0–87.0.0	21.2–41.8.2.8
DASH Reverse	23	68.5 ± 23.8	69.0	13–100	58.2–78.8
EFAS	26	64.3 ± 22.3	67.0	5–100	55.3–73.3

#### Soft-tissue defects and reconstruction

Among the 43 mandibular cases, soft-tissue involvement was most frequent in the floor of mouth (67.4%), buccal mucosa (44.2%), tongue (32.6%), and external skin (16.3%). Lateral mandibular defects were most often associated with floor-of-mouth and buccal mucosa involvement, while latero-central defects correlated with tongue resections, and latero-central-lateral defects with skin loss (Tables 5 and 6).

**Table 5:** SHI Scores: Fibula vs. Scapula

	Fibula (*N* = 26)	Scapula (*N* = 23)	*p* value
SHI			0.561
Median (IQR)	32.5 (9–59)	24 (7.50–45)	
Range	0.0–100.0	0.0–65.0

**Table 6:** MDADI Scores: Fibula vs. Scapula

	Fibula (*N* = 24)	Scapula (*N* = 22)	*p* value
Global score			0.964
Median (IQR)	4 (2–5)	4 (3–5)	
Range	1.0–5.0	1.0–5.0	
Composite score			0.860
Median (IQR)	72.5 (57.8–85.8)	72.5 (60–83)	
Range	42.0–99.0	39.0–100.0	
Emotional score			0.956
Median (IQR)	77 (56.8–87.8)	72 (58.8–90)	
Range	34.0–100.0	37.0–100.0	
Functional score			0.895
Median (IQR)	82 (60–96)	78 (72–87)	
Range	24.0–100.0	36.0–100.0	
Physical score			0.659
Median (IQR)	67.5 (55–83)	68 (55.8–77.3)	
Range	43.0–100.0	38.0–100.0	

Soft-tissue reconstruction varied by flap type. In the fibula group, the peroneal skin paddle was used in 81.1%, sometimes combined with an ALT flap (16.2%). In the scapular group, soft-tissue coverage was achieved with TDAP + latissimus dorsi (46.9%), TDAP + serratus anterior (40.6%), or serratus alone (12.5%). Only one scapular case (3.1%) required no additional soft-tissue flap (Table 7).

**Table 7:** Quality of life assessment with the EORTC QLQ-H&N35

	Fibula (*N* = 37)	Scapula (*N* = 32)	*p* value
Pain, median (IQR)	17 (9–42)	25 (9–58.5)	0.495
Sense problems	17 (0–34)	50 (0–58.5)	0.193
Trouble with social eating	29.5 (11–73)	42 (25–54.5)	0.920
Trouble with social contact	27 (8.75–40)	34 (14–53.5)	0.763
Teeth	17 (0–34)	0 (0–67)	0.982
Opening Mouth	50.5 (0–100)	67 (34–100)	0.393
Dry Mouth	34 (0–100)	67 (34–100)	0.122
Sticky saliva	34 (0–67)	34 (0–83.5)	0.884
Nutritional supplements			0.195
No	15 (57.7%)	9 (39.1%)	
Yes	11 (42.3%)	14 (60.9%)	
Weight loss			0.208
No	22 (84.6%)	16.0 (69.6%)	
Yes	4 (15.4%)	7.0 (30.4%)	
Weight gain			0.551
No	19 (73.1%)	15 (65.2%)	
Yes	7 (26.9%)	8 (34.8%)	

#### Postoperative outcomes

Median time to oral feeding was similar between groups (fibula: 15.5 days, IQR 12–21.8; scapula: 15 days, IQR 11–24.5); four patients did not resume oral intake during hospitalization. Median decannulation time was 9 days (IQR 7–11) for fibula and 7 days (IQR 6–14) for scapula; 11 cases were missing due to absence of tracheostomy or delayed decannulation. Median hospital stay was 21 days (IQR 17–26) in the fibula group and 23 days (IQR 17–33) in the scapula group, with no significant difference (*p* = 0.643) (Table 8).

**Table 8:** FACE-Q appearance module scores: Fibula vs. Scapula

	Fibula (*N* = 26)	Scapula (*N* = 23)	*p* value
FACE - Q Appearance			0.532
Mean ± SD	53.7 ± 24.1	51.2 ± 25.6	
Range	12.0–100.0	20.0–100.0	

### Complications

#### In-hospital complications

Postoperative complications are summarized in Table 9. Complications were categorized as early or late, and as medical or surgical; several patients had multiple events. Early complications occurred in 18.8% of cases, more frequently in the scapula group (28.1%) than the fibula group (10.8%), without statistical significance (*p* = 0.067). Late complications were reported in 71.0% of patients, with similar distribution (fibula 67.6%, scapula 75.0%).

**Table 9:** Site-stratified complications (mandible-only vs. maxilla-only)

	Mandible-only			Maxilla-only		
	Fibula (*N* = 32)	Scapula (*N* = 10)	*p* value	Fibula (*N* = 5)	Scapula (*N* = 21)	*p* value
Short onset complications	4 (12.5%)	1 (10%)	^1^	0 (0%)	7 (33.3%)	0.278
Late onset complications	22 (68.8%)	7 (70.0%)	1	3 (60.0%)	15 (71.4%)	0.628
Surgical complications	16 (50.0%)	6 (60.0%)	0.723	1 (20.0%)	15 (71.4%)	0.055
Flap Failure	7 (21.9%)	0	0.168	0 (0%)	6 (28.6%)	0.298
Donor site complications	9 (28.1%)	0 (0%)	^0.091^	1 (20.0%)	1 (4.8%)	0.354
Medical complications	11 (34.4%)	5 (50%)	0.465	1 (20.0%)	4 (19.0%)	1.000
Long term complications	7 (21.9%)	4 (0%)	0.256	2 (40.0%)	9 (42.9%)	1.000
Plate/bone exposure	4 (12.5%)	3 (30.0%)	0.332	1 (20.0%)	7 (33.3%)	1.000
Fistula	3 (9.4%)	2 (20.0%)	0.424	2 (40.0%)	5 (23.8%)	0.588
Osseous nonunion	1 (3.1%)	0 (0%)	1.000	0 (0%)	1 (4.8%)	1.000
Osteoradionecrosis	0 (0%)	0 (0%)		0 (0%)	1 (4.8%)	1.000
Recipient site infection	1 (3.1%)	0 (0%)	1.000	0 (0%)	1 (4.8%)	1.000
Any revision surgery	21 (65.6%)	2 (20.0%)	0.026	3 (60.0%)	8 (38.1%)	0.624
Urgent revision	5 (15.6%)	2 (20.0%)	1.000	0 (0%)	5 (23.8%)	0.182
Elective revision	20 (62.5%)	2 (20.0%)	^0.343^	3 (60.0%)	6 (28.6%)	0.329

Surgical complications affected 56.5% of patients, more often in the scapula cohort (68.8% vs. 45.9%; *p* = 0.057). Flap failure (partial or total) occurred in 20.2%: 13 partial necroses (18.8%; fibula 7, scapula 6) and one total loss (1.4%, scapula). Donor-site morbidity was significantly higher in the fibula group (24.3% vs. 6.2%; *p* = 0.041). Medical complications occurred in 30.4% of patients (fibula 32.4%, scapula 28.1%), including cardiovascular events (4.3%), respiratory issues (10.1%), and other systemic complications—infective, renal, or metabolic (23.2%) (Table 10).

**Table 10:** Site-stratified PROMS (mandible-only vs. maxilla-only)

	Mandible-only PROMs			Maxilla-only PROMs		
	Fibula	Scapula	*p* value	Fibula	Scapula	*p* value
SHI	24	5	^0.908^	2	17	0.096
MDADI						
Composite	22	4	0.319	2	17	0.183
Global	22	4	0.464	2	17	0.575
EORTC QLQ-H&N35						
Pain	24	5	0.277	2	17	0.685
Sense problem	24	5	0.715	2	17	0.118
Social eating	24	5	0.157	2	17	0.052
Social contact	24	5	0.465	2	17	0.129
Teeth	24	5	0.199	2	17	0.956
Opening mouth	24	5	0.421	2	17	0.070
Dry mouth	24	5	0.090	2	17	0.071
Sticky saliva	24	5	0.571	2	17	0.111
FACE-Q Appearance	19	4	0.467	2	17	0.072

#### Donor-site complications: definition, classification, and management

Because of the retrospective design of this study, donor-site complications were recorded from clinical charts as binary events (present/absent) rather than graded prospectively according to severity-based classification systems. Complications were identified through postoperative inpatient notes, outpatient follow-up documentation, and surgical reports. Although formal severity grading was not feasible, donor-site morbidity was further characterized descriptively to improve clinical interpretability.

In the fibula free flap cohort, donor-site complications included wound dehiscence, delayed wound healing, local infection, and transient gait disturbance. Wound-related complications typically consisted of partial skin dehiscence or marginal necrosis at the donor site and were managed conservatively with local wound care, dressing changes, and, when indicated, short courses of oral antibiotics. No patient required surgical re-exploration of the lower limb. Gait disturbance was documented in a limited number of patients and was transient, resolving with physiotherapy and gradual mobilization during follow-up.

In the scapular free flap cohort, donor-site morbidity was less frequent and primarily consisted of mild shoulder dysfunction and transient sensory disturbances. Shoulder dysfunction manifested as reduced range of motion or stiffness and was managed with structured physiotherapy. Sensory symptoms were consistent with transient neuropraxia and resolved spontaneously without surgical interventi.

on. No cases of chronic shoulder instability or permanent neurologic deficit were observed. Across both cohorts, no donor-site complication resulted in long-term functional impairment, need for reoperation, or permanent disability. These findings are consistent with the patient-reported outcome measures, which demonstrated preserved long-term limb function despite the presence of early donor-site morbidity.

#### Post-discharge complications

Long-term complications occurred in 18 patients (26.1%), more often in the scapula group (31.2%) than the fibula group (21.6%), without statistical significance (*p* = 0.364). The most frequent events were plate/bone exposure (12 cases, 17.4%; fibula 13.5%, scapula 21.9%) and persistent fistula (9 cases, 13.0%; scapula 18.8%, fibula 8.1%). Pseudoarthrosis occurred in 2 patients (2.9%), osteoradionecrosis in 1 scapula case, and recurrent infection in 2 cases (equal distribution). No individual complication showed a significant intergroup difference.

#### Surgical revisions

Elective surgical revisions were more frequent in the fibula group (62.2% vs. 31.3%; *p* = 0.010). Indications for revision procedures differed according to reconstructive goals and included three main categories: implant-driven procedures, hardware-related revisions, and aesthetic or contouring refinements.

Implant-driven revisions were primarily related to preparation for dental rehabilitation, including plate adjustments or bone contouring to facilitate implant placement. Hardware-related revisions included management of plate exposure or hardware irritation. Aesthetic revisions involved secondary contouring procedures aimed at improving mandibular symmetry or soft-tissue profile.

In the fibula cohort, a considerable proportion of elective revisions were associated with staged implant-related procedures, reflecting the frequent use of the fibular flap for mandibular reconstruction and its suitability for osseointegrated dental rehabilitation.

### Patient-reported outcome measures (PROMs)

Functional, aesthetic, and quality-of-life outcomes were assessed using validated PROMs at least 6 months postoperatively, ensuring stabilization of the postoperative clinical status. Of the 69 patients, 49 (71.0%) completed questionnaires. Fourteen patients (20.3%) had died prior to data collection, 5 patients (7.2%) declined participation, and 1 patient (1.4%) was unreachable.

PROMs collected included:

- Upper limb function: DASH(scapula flaps) and EFAS (fibula flaps) scores showed no significant intergroup differences (*p* > 0.05). However, patients with extended scapular harvests (including the lateral border) reported increased shoulder stiffness and reduced range of motion, in line with literature recommendations for targeted physiotherapy. DASH_reverse analysis showed significantly lower scores in combined TDAP + serratus harvests (55.5 ¬± 24.8) compared with TDAP (80.6 ¬± 15.0) or serratus alone (80.0 ¬± 21.7; *p* = 0.034).
- Speech function: SHI scores indicated similar levels of speech handicap across groups. Forty-nine patients completed the SHI (fibula *n* = 26, scapula *n* = 23). Median scores were 32.5 (IQR 7–47) overall, with no significant differences between groups (fibula 32.5 vs. scapula 24.0; *p* > 0.05).
- Swallowing function: MDADI was completed by 46 patients (fibula *n* = 24, scapula *n* = 22; 3 PEG patients excluded). Global scores were similar (median 4 in both groups). Composite medians were 72.5 in both groups, with no intergroup differences across emotional, functional, or physical subscales (all *p* > 0.05), with comparable recovery profiles in both cohorts.
- Quality of life & aesthetics: EORTC QLQ-H&N35 and FACE-Q Appearance scores demonstrated similar patient-perceived outcomes between fibula and scapula flaps: Pain, sensory disturbances, and social difficulties were reported at comparable levels in both groups, with no significant differences. Some trends were noted: higher sensory disturbances and worse oral dryness in scapula reconstructions, while fibula patients more often reported dental problems. No variable reached statistical significance.

Overall, despite anatomical and reconstructive differences, both fibula and scapula flaps achieved excellent long-term functional and aesthetic outcomes when appropriately indicated.

### Site-stratified analyses (mandible-only vs. maxilla-only)

#### Site-stratified complications (mandible-only vs. maxilla-only)

We performed additional site-stratified analyses to address potential confounding due to the different distribution of flap types across mandibular and maxillary reconstructions. The single patient with combined maxilla–mandible involvement was excluded from stratified testing. Within each anatomical subgroup, complications were compared using Fisher’s exact test, and PROMs using Mann–Whitney U tests due to small subgroup sizes.

In the mandible-only subgroup (Fibula *n* = 32, Scapula *n* = 10), overall complication rates were comparable between flap types; however, any revision surgery was significantly more frequent after fibula reconstruction (65.6% vs. 20.0%, *p* = 0.026). In the maxilla-only subgroup (Fibula *n* = 5, Scapula *n* = 21), no statistically significant differences were observed for complications; a trend toward higher surgical complication rates in scapula reconstructions did not reach significance (71.4% vs. 20.0%, *p* = 0.055) (Table 10).

#### Site-stratified PROMS (mandible-only vs. maxilla-only)

Site-stratified PROM comparisons did not demonstrate significant differences between flap types in either subgroup, although interpretation in the maxilla-only fibula cohort is limited by small PROM completion (Table 10).

## Discussion

This study compares fibular and scapular osseous flaps in cervicofacial reconstruction with respect to clinical outcomes, donor-site morbidity, and patient-reported quality of life. The fibula flap remains the reference option for mandibular defects requiring long bone segments and dental rehabilitation [17]. In our cohort, it was mainly used for mandibular reconstruction, frequently with CAD/CAM plates, but showed more elective revisions, likely reflecting the challenges of restoring occlusion and contour. The scapular flap was preferentially selected for maxillary defects and in patients with vascular comorbidities contraindicating fibula harvest [18, 19], Its donor-site morbidity was significantly lower (6.3% vs. 24.3%), corroborating reports of its safer profile, and its soft-tissue versatility facilitates midface contouring and orbital floor support [20]. Functional outcomes were comparable: MDADI, SHI, EORTC QLQ-H&N35, and FACE-Q scores overlapped between groups, consistent with prior studies [12]. Donor-site function was largely preserved, although extended scapular harvest reduced shoulder mobility, underscoring the role of physiotherapy. While the fibula remains superior for long mandibular spans and osseointegrated implants, its use is limited in patients with peripheral vascular disease [18]. The scapular flap, although less suitable for very long defects, provides reliable bone segments with abundant soft tissue for lining or resurfacing [19, 20]. Increasing use of CAD/CAM planning has reduced technical disparities, improving accuracy and operative efficiency regardless of flap choice [17, 21]. Study limitations include its retrospective design, absent data on long-term implant rehabilitation, and possible selection bias. Overall, findings support the scapular flap as a safe, functionally equivalent, and aesthetically adaptable alternative to fibula in appropriately selected patients.

### Clinical-demographic characteristics

The groups differed significantly in median age: scapula patients were older (63.0 years) than fibula patients (53.5 years), consistent with reports identifying the scapula as preferable in older, frailer individuals. Fibula harvest may be contraindicated in vascular anomalies or tibial atherosclerosis, which affects up to 25% of elderly patients [18]. Scapular vessels show minimal atherosclerotic involvement, maintaining reliability even in peripheral arterial disease [19]. Earlier postoperative mobilization is another advantage in elderly patients, decreasing risks of pneumonia, thromboembolism, and muscle atrophy. Follow-up duration was longer in the fibula cohort, representing a potential bias in long-term comparisons. Other baseline variables (sex, smoking, comorbidities, indication) were comparable.

#### Surgical aspects

**Fig. 1:**
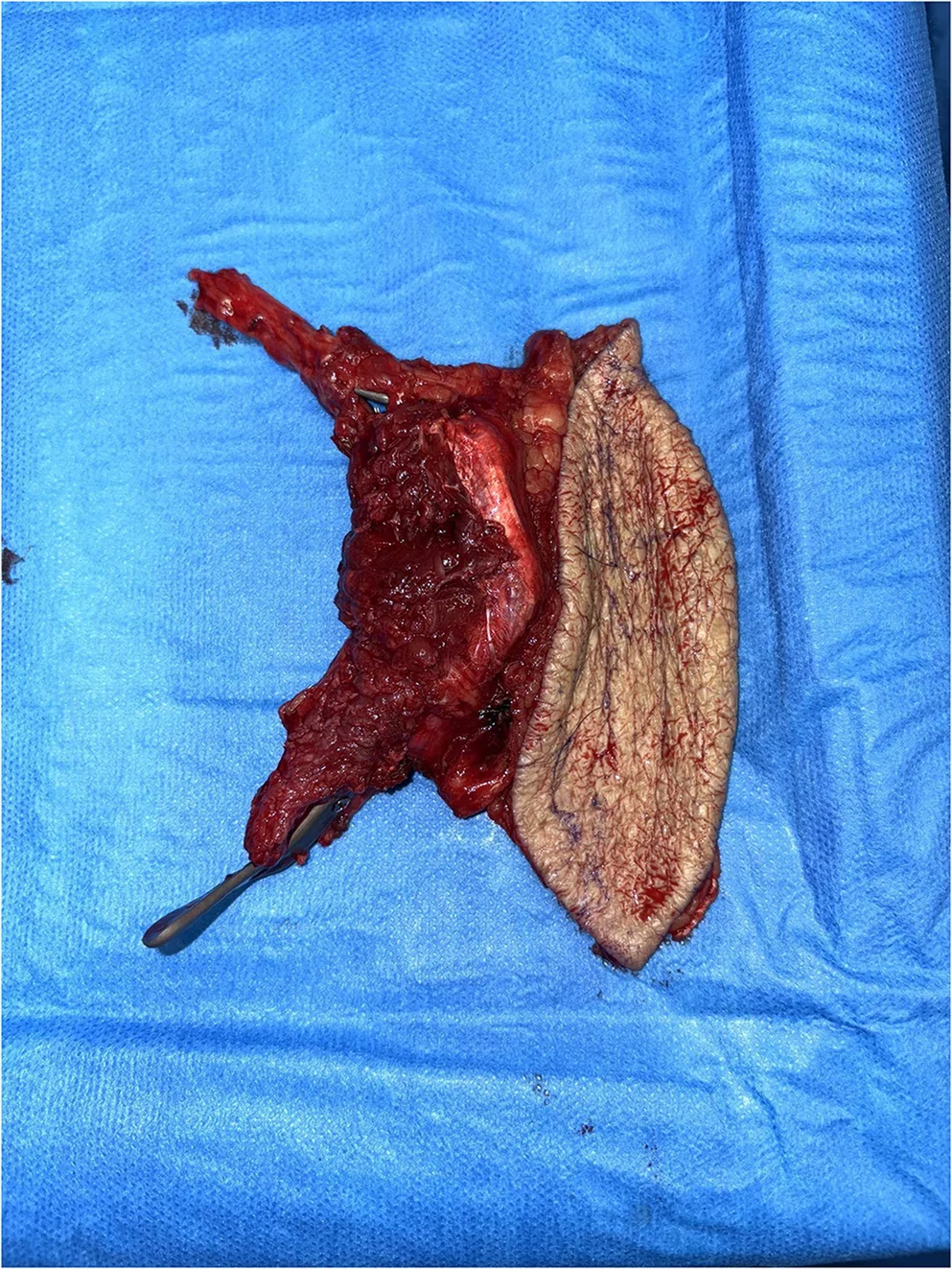
Left. Intraoperative view of the mandibular Custom-made CAD/CAM plate and the fibular flap

**Fig. 2:**
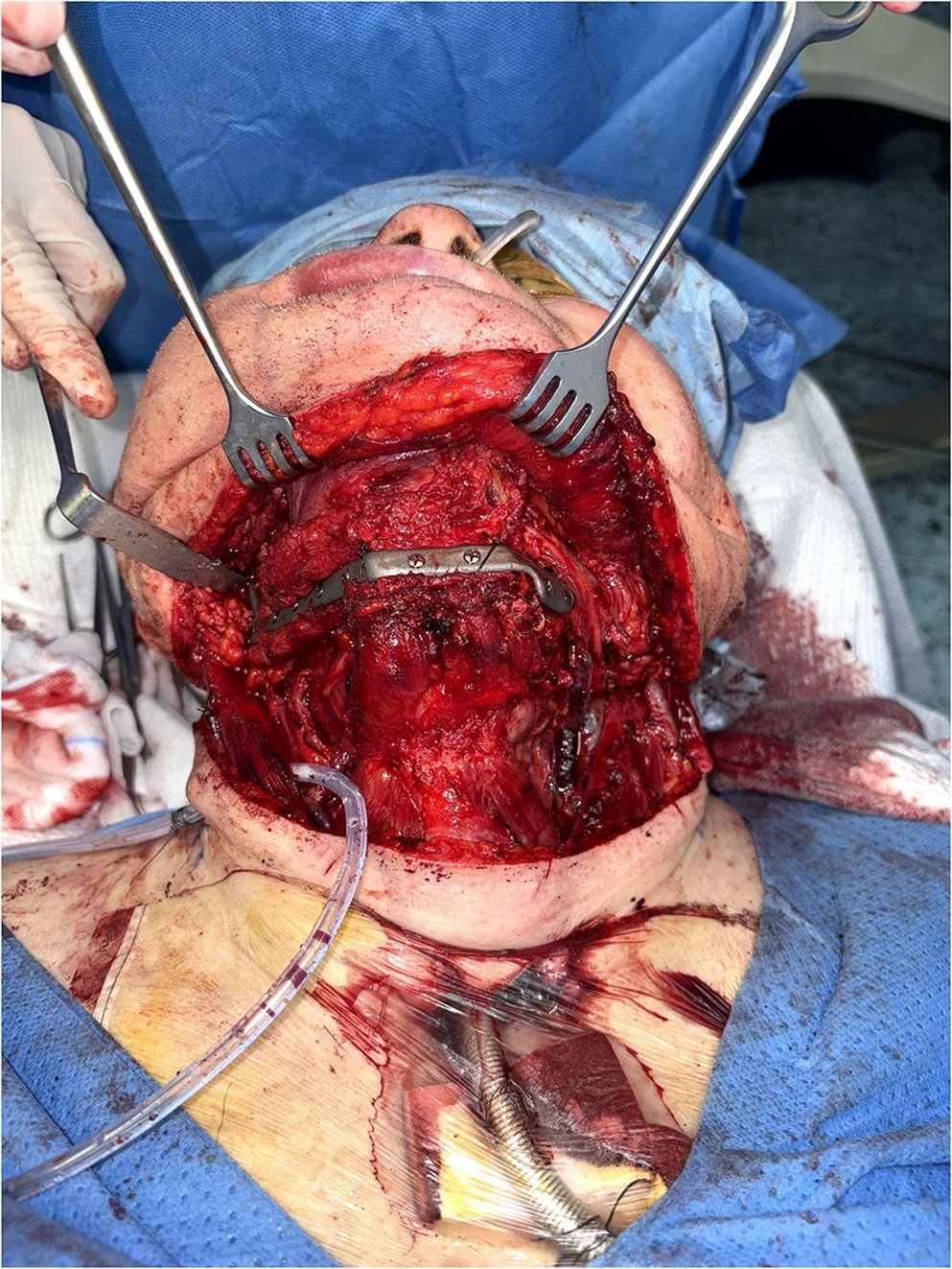
Right. Insetting of the mandibular plate and the fibular flap

**Fig. 3:**
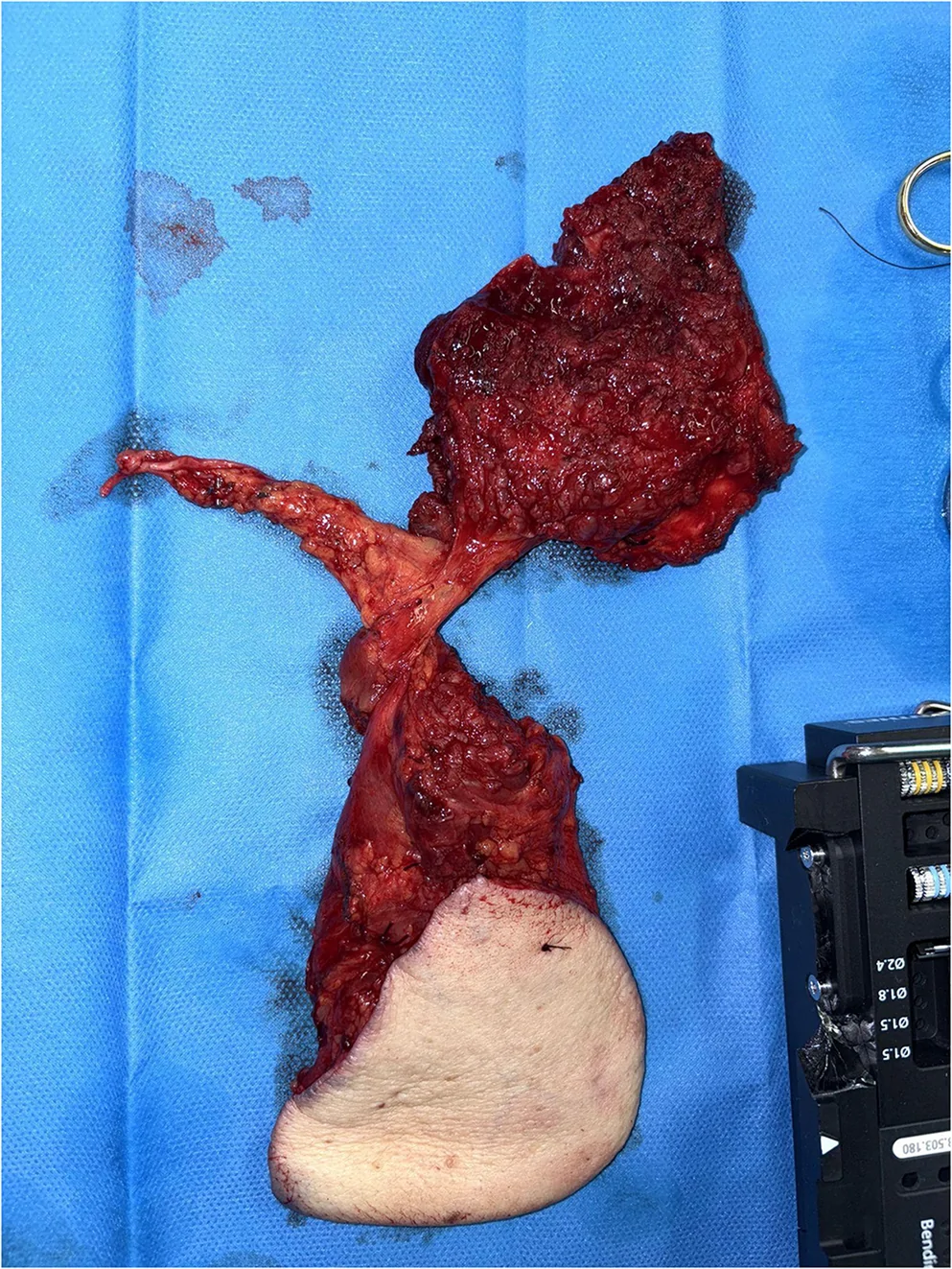
Left. Intraoperative view of the scapula flap

**Fig. 4:**
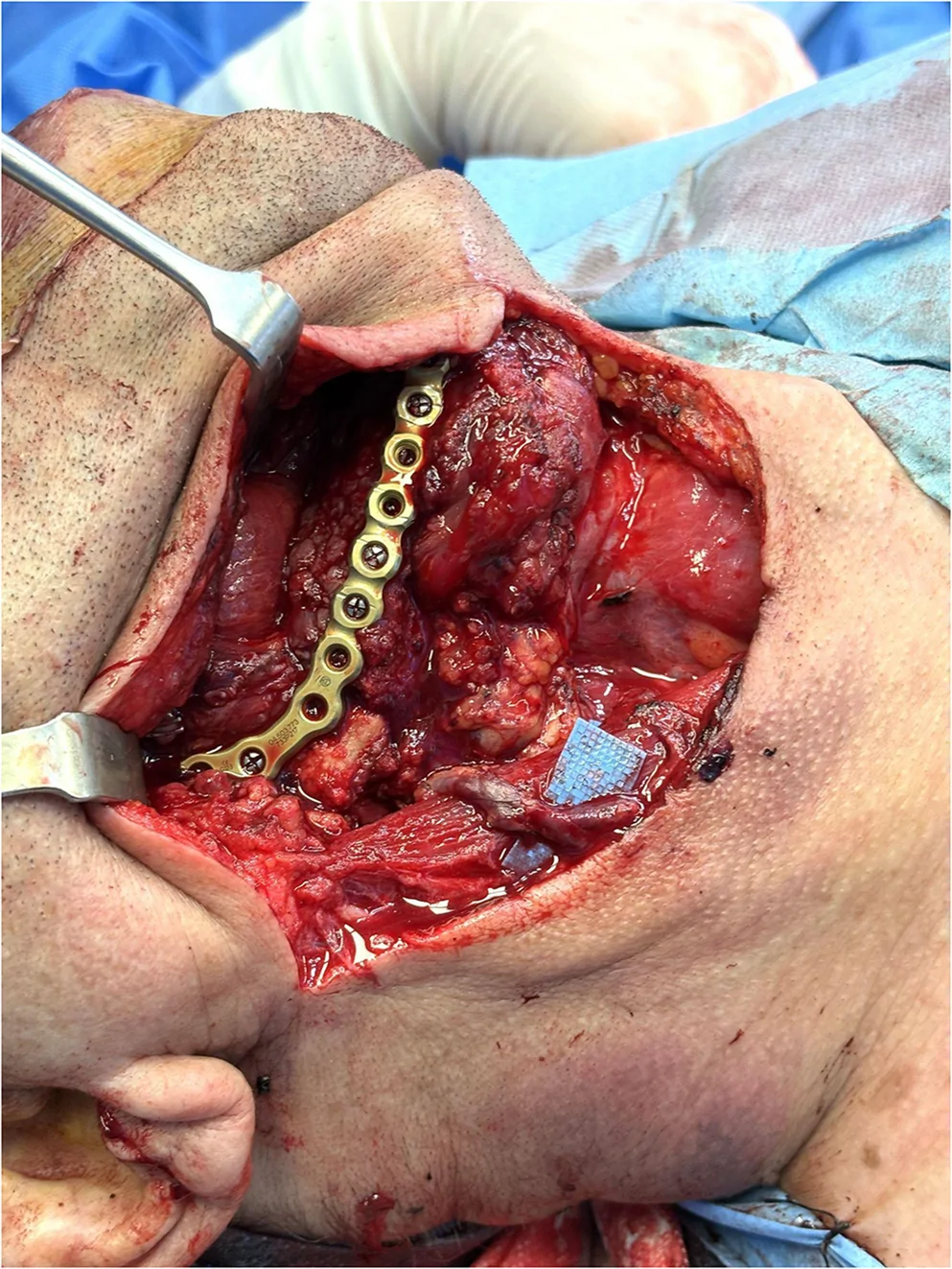
Right. Insetting of the mandibular plate and the scapula flap

**Fig. 5:**
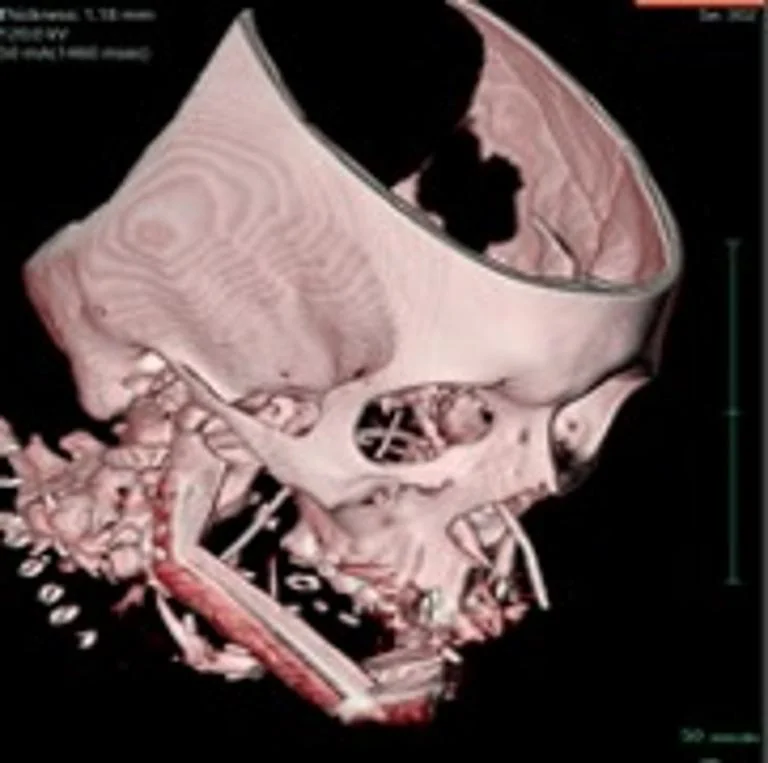
3D Post-operative CT Reconstruction showing the mandibular reconstruction

Fibula was predominantly used for mandibular reconstruction, scapula for maxillary defects, aligning with current recommendations [12, 18]. Fibula provides long, high-quality bone with multiple osteotomy options, whereas scapula offers greater soft-tissue versatility [20]. Custom CAD/CAM plates were significantly more frequent in fibula cases, reflecting wider adoption of virtual planning, which enhances precision and shortens intraoperative modeling but increases cost and logistical complexity [21]. Operative time was similar between groups (11.7 h fibula vs. 11.9 h scapula), consistent with some studies [18, 22], although others report shorter fibula times, attributed to two-team approaches [23–25]. This suggests that operative efficiency depends more on surgical workflow and experience than flap type (Figs. 1, 2, 3, 4 and 5).

#### Hospital stay

No significant differences were observed in recovery endpoints (time to oral feeding, decannulation, length of stay), consistent with Liu et al. and other comparative studies [23–25]. Early/late complication rates and flap failure incidence were also similar, confirming both flaps as safe and reliable options [18, 25]. Donor-site morbidity, however, differed significantly, with higher rates in fibula patients, reinforcing the scapular flap’s safer profile [18, 19].

#### Donor-site complications

Fibula harvest resulted in more local adverse events (24.3%), including seroma, hematoma, dehiscence, and delayed healing, whereas scapular morbidity was lower (6.2%) and generally mild. This aligns with literature citing donor-site morbidity as a major limitation of the fibula flap. Reported complication rates vary [26, 27] but remain consistently higher than for scapula [22]. A systematic review of 24 studies reported mean donor-site morbidity of 31.2% for fibula versus 10.7% for scapula, confirming a clinically relevant difference [28]. Use of skin grafts for fibula closure increases delayed healing risk [29], prompting refinements such as minimizing paddle size to allow primary closure [30]. Scapular harvest typically permits direct closure with favorable vascularity and reduced mechanical stress, explaining its lower morbidity [31]. Early limb mobilization and physiotherapy further support healing in scapula patients [28].

#### Post-discharge complications

Analysis of late complications included plate/bone exposure, persistent fistula, pseudoarthrosis, osteoradionecrosis, and recurrent infection, with no significant differences between flaps. Literature remains conflicting: Tsang et al. [32] reported higher plate exposure in fibula mandibular reconstructions, attributed to limited soft-tissue coverage, variable skin vascularity, and increased infection risk compared with scapula [32]. A recent multicenter study confirmed greater long-term risk in fibula cases [33]. Other authors found comparable rates, suggesting that chemoradiotherapy, defect site, and soft-tissue management may influence late morbidity more than flap type [34].

#### Surgical revisions

Reoperation rates were significantly higher in the fibula group; however, interpretation of this finding requires contextualization. Elective revisions in this cohort were frequently implant-driven procedures related to dental rehabilitation planning, including contouring of the reconstructed mandible or adjustments of fixation hardware to facilitate implant placement. These staged procedures reflect the favorable osseous characteristics of the fibula, which make it particularly suitable for osseointegrated dental implants and functional occlusal rehabilitation.

Additional revisions were related to hardware-associated issues, such as plate exposure or irritation, as well as secondary aesthetic contouring procedures aimed at improving mandibular symmetry and soft-tissue profile. Importantly, many of these interventions represent planned reconstructive refinements rather than complications of the primary reconstruction.

Furthermore, the longer follow-up duration observed in the fibula cohort may have increased the likelihood of detecting late implant-related procedures and patient-driven aesthetic refinements, potentially contributing to the observed difference in revision rates.

### Patient-reported outcomes (PROs)

#### Donor-site functional assessment

Donor-site morbidity was evaluated using EFAS (fibula) and DASH (scapula). Both demonstrated good residual limb function, though not full normality. Mean EFAS was 64.3 (± 22.3 SD), consistent with satisfactory lower-limb function, although comparison is limited by heterogeneous scales in the literature [22, 25]. Despite higher early complication rates, long-term functional impact after fibula harvest is generally minimal [35]. It should be noted that the DASH and EFAS instruments assess different anatomical regions and functional domains, reflecting the distinct donor sites of the two flaps. The DASH questionnaire evaluates upper-limb function and disability related to shoulder and arm movement following scapular flap harvest, whereas the EFAS score assesses pain and functional performance of the foot and ankle after fibular harvest. Consequently, these instruments are not directly interchangeable and were not intended for direct quantitative comparison between cohorts. Instead, each PROM provides a contextual evaluation of donor-site functional outcomes specific to the anatomical region involved in flap harvest.

For scapula, mean DASH was 31.5 (± 23.8 SD), higher than the U.S. normative mean of 10.1 (± 14.7) [36] and classified as “problem, but working” [16–40] in Kennedy et al.’s grading system [37], indicating preserved activities of daily living despite mild impairment. Slightly worse values than reported elsewhere may reflect surgical technique, sample characteristics, and short follow-up [median 7.5 months], which may underestimate late recovery. Neck dissection likely contributed to shoulder dysfunction [38], and age-related decline also increases DASH values in individuals > 50 years [39]. Stratified analysis showed lower scores in TDAP + serratus harvests, consistent with greater dissection of stabilizing musculature. Overall, donor-site limitations were modest in both flaps and compatible with good long-term quality of life, consistent with published data.

#### Speech, swallowing, quality of life, and facial aesthetics

No significant differences were detected between groups for SHI, MDADI, EORTC QLQ-H&N35, or FACE-Q Appearance scores, in line with prior comparative studies. Liu et al. reported no UW-QOL differences between fibula and scapula reconstructions [25]. Dimovska et al. [22] using FACE-Q Head & Neck Cancer scales also found overall equivalence, though scapula showed a trend toward better Appearance and Eating/Drinking scores in lateral and centrolateral mandibular defects [22]. In our stratified analysis by defect site [lateral/centrolateral mandibulectomy, maxillectomy], no significant differences emerged. A 2024 meta-analysis of 22 studies identified age, radiotherapy, tumor stage, and dysphagia—not flap type—as the main predictors of poorer QoL [40]. Thus, patient-reported outcomes reflect multifactorial influences beyond the choice of osseous flap.

## Conclusions

Within the limitations of this retrospective single-center study, both fibular and scapular osseous free flaps demonstrated favorable reconstructive outcomes in cervicofacial surgery. The fibular flap remains widely used for mandibular reconstruction requiring long bone segments and dental rehabilitation, while the scapular flap represents a reliable alternative, particularly in patients with vascular comorbidities or when extensive soft-tissue reconstruction is required. These findings support the role of individualized flap selection based on anatomical, functional, and patient-related factors.

## Key findings

1. **Patient selection** – Comparable success rates support individualized flap choice based on anatomical, functional, and systemic factors rather than institutional routine.
2. **Donor-site morbidity** – Significantly lower after scapular harvest, with faster recovery and reduced rehabilitation needs, consistent with published evidence.
3. **Patient-reported outcomes** – Speech, swallowing, limb function, aesthetics, and quality of life were similar, confirming satisfactory restoration with both flaps.
4. **Revisions** – More frequent in the fibula cohort, likely related to longer follow-up.
5. **Technology** – Increasing CAD/CAM use may improve precision and expand indications for scapular reconstruction.

The scapular flap should therefore be considered an important reconstructive option with specific advantages in selected clinical scenarios. However, prospective multicenter studies with standardized long-term follow-up, including evaluation of implant rehabilitation and functional recovery, are needed to further clarify optimal flap selection and long-term outcomes.
